# Morphometric Analysis of Sexual and Lateral Dimorphism in the Anterior Border of the Human Hip Bone: Implications for Anatomical Variation and Clinical Applications

**DOI:** 10.7759/cureus.97812

**Published:** 2025-11-25

**Authors:** Deepak Sharma, Prakrati Dr, Shiv Prakash Sharma, Shatrughan Kumar

**Affiliations:** 1 Anatomy, Rajasthan University of Health Sciences (RUHS) College of Medical Sciences, Jaipur, IND; 2 Preventive Medicine, Rajasthan University of Health Sciences (RUHS) College of Medical Sciences, Jaipur, IND

**Keywords:** anatomical variation, bilateral asymmetry, forensic anthropology, osteometry, pelvic bones, surgical anatomy

## Abstract

Introduction: A comprehensive understanding of the normal morphometric variation of the hip bone is crucial for both anatomical science and clinical practice. While sexual dimorphism is well-established, detailed analysis of bilateral asymmetry and its interaction with sex in the anterior pelvic region remains less explored. This study aims to conduct a detailed morphometric analysis of the anterior hip border, quantifying both sexual and lateral dimorphism.

Methods: An observational study was conducted on 88 adult human hip bones (44 male, 44 female), with equal representation from right and left sides. Eight linear parameters of the anterior border were measured using digital Vernier calipers. Data were analyzed using IBM SPSS Statistics for Windows, Version 23.0 (IBM Corp., Armonk, New York, United States). Sexual dimorphism was assessed with independent samples t-tests, while lateral dimorphism (right-left asymmetry) was evaluated using paired samples t-tests within each sex. A two-way analysis of variance (ANOVA) was employed to investigate the interaction between sex and side.

Results: Significant sexual dimorphism was observed in all eight parameters (p<0.001 for all), with male bones exhibiting larger mean dimensions. Analysis of lateral dimorphism revealed no statistically significant difference between the right and left sides in either males or females for any of the measured parameters (p>0.05 for all). The two-way ANOVA confirmed a significant main effect of sex (p<0.001) but no significant main effect of side or sex-side interaction, indicating that the observed dimorphism is driven solely by sex without notable bilateral asymmetry.

Conclusion: The anterior border of the hip bone exhibits pronounced and consistent sexual dimorphism without significant lateral asymmetry. This consistency between sides reinforces the reliability of these metric parameters for applications requiring bilateral comparison, such as preoperative surgical planning for procedures like total hip arthroplasty or pelvic osteotomy, and in forensic identification. The findings provide a robust baseline of normal anatomical variation for the North Indian population.

## Introduction

The identification of sex from human skeletal remains is a cornerstone of forensic anthropology and bioarchaeological inquiry. Among all skeletal elements, the hip bone, or os coxae, is universally regarded as the most reliable indicator due to its pronounced sexual dimorphism, which is fundamentally linked to functional adaptations for parturition in females [1]. These morphological differences are so distinct that sex can often be determined through the visual assessment of traits such as the greater sciatic notch, the subpubic angle, and the preauricular sulcus [2]. However, non-metric or morphological methods are inherently subjective, and their accuracy is heavily dependent on the observer's expertise and experience [3]. In contrast, osteometric analysis provides an objective, reproducible, and statistically verifiable alternative, reducing observer bias and allowing for the calculation of classification probabilities [4].

Extensive research has established the utility of various pelvic measurements for sex estimation, with methods ranging from single measurements to complex multivariate models [4,5]. While the sciatic notch and pubis have been traditional foci, the specific morphological complex of the anterior border, encompassing landmarks like the anterior superior iliac spine (ASIS), anterior inferior iliac spine (AIIS), pubic tubercle (PT), and symphysial surface (SS), has received comparatively less attention. This region is particularly valuable as it is robust and often well-preserved in forensic contexts. Pioneering work by Gómez Pellico and Fernández Camacho first systematically documented the biometry of this area, demonstrating its value for sex determination [6]. Subsequent studies, such as that by Sachdeva et al. in a North Indian population, confirmed the significant sexual dimorphism present in these anterior border metrics [7]. Despite this, there remains a pressing need for population-specific standards due to well-documented geographical and ethnic variations in skeletal anatomy [8,9].

The North Indian population represents a large demographic for which contemporary, statistically robust osteometric standards are still developing. Therefore, this study aims to develop and validate a population-specific discriminant function model for sex determination using the metric analysis of the anterior border of the hip bone. The primary objective is to identify the most dimorphic parameters and combine them into a practical mathematical formula that can be applied with high accuracy in forensic casework within this region.

## Materials and methods

Study design and sample

This observational study utilized a sample of 88 dry, adult human hip bones of known sex. The sample comprised 44 bones from males and 44 from females, with an equal distribution of right-sided (n=44) and left-sided (n=44) specimens within each sex group. The bones were obtained from the Department of Anatomy at Rajasthan University of Health Sciences (RUHS) College of Medical Sciences, Jaipur, India, and Sawai Man Singh (SMS) Medical College, Jaipur, India. The inclusion criteria were intact bones with no macroscopic evidence of pathological deformity or traumatic insult. Specimens exhibiting osteoarthritic changes, fractures, or any other damage that could distort the anatomical landmarks were excluded. Ethical approval for the study was granted by Rajasthan University of Health Sciences (RUHS) College of Medical Sciences Ethics Committee (approval number: 415).

Data collection

Eight metric parameters of the anterior border were measured on each specimen using a digital Vernier caliper with an accuracy of 0.1 mm. The measurements, based on established protocols, included straight-line distances between the following landmarks: the anterior superior iliac spine to the symphysial surface (ASIS-SS), the anterior superior iliac spine to the pubic tubercle (ASIS-PT), the anterior inferior iliac spine to the symphysial surface (AIIS-SS), the anterior inferior iliac spine to the pubic tubercle (AIIS-PT), the anterior inferior iliac spine to the iliopubic eminence (AIIS-IE), and the iliopubic eminence to the pubic tubercle (IE-PT). Additionally, two arch distances were recorded: from the anterior superior iliac spine to the symphysial surface (Arch ASIS-SS) and from the anterior inferior iliac spine to the iliopubic eminence (Arch AIIS-IE). All measurements were performed by a single investigator to ensure consistency and minimize inter-observer variability.

Statistical analysis

Statistical analysis was conducted using IBM SPSS Statistics for Windows, Version 23.0 (IBM Corp., Armonk, New York, United States). Descriptive statistics (mean and standard deviation) were calculated for all parameters, stratified by sex and side. The analysis proceeded in two stages. First, sexual dimorphism was assessed using an independent samples t-test, comparing all measurements between the pooled male and female groups. Second, lateral dimorphism was evaluated using a paired samples t-test, comparing right-side and left-side measurements within the male and female groups separately. Finally, a two-way analysis of variance (ANOVA) was employed to examine the main effects of sex and side, as well as their interaction effect, on each measurement. A p-value of less than 0.05 was considered statistically significant for all tests.

## Results

The descriptive statistics for all measurements, presented by sex and side, are detailed in Table 1. The independent samples t-test revealed that all eight parameters exhibited highly significant sexual dimorphism, with male specimens demonstrating consistently larger mean values than female specimens (p<0.001 for all comparisons).

**Table 1:** Descriptive statistics (mean±SD in mm) of anterior border measurements stratified by sex and side The p-value for sex is derived from an independent samples t-test comparing all males vs. all females. SD: standard deviation; ASIS: anterior superior iliac spine; AIIS: anterior inferior iliac spine; PT: pubic tubercle; SS: symphysial surface; IE: iliopubic eminence

Measurement	Males (n=44)		Females (n=44)		P-value (sex)
	Right (n=22)	Left (n=22)	Right (n=22)	Left (n=22)	
ASIS-SS	133.5±3.1	132.3±3.4	129.6±1.9	129.2±2.7	<0.001
ASIS-PT	120.0±3.3	118.6±2.8	114.6±1.3	114.0±2.1	<0.001
AIIS-SS	101.3±2.6	100.4±2.9	98.2±1.4	98.0±1.5	<0.001
AIIS-PT	87.2±1.8	86.6±1.7	82.4±2.1	82.0±2.0	<0.001
AIIS-IE	39.2±1.8	38.8±1.6	37.1±1.3	36.9±1.2	<0.001
IE-PT	50.6±1.6	50.5±1.5	49.2±1.7	49.6±1.6	0.001
Arch ASIS-SS	158.1±1.3	157.5±1.4	155.2±1.6	155.4±1.5	<0.001
Arch AIIS-IE	43.1±1.5	42.9±1.4	40.8±1.5	40.7±1.4	<0.001

The analysis of lateral dimorphism, assessed via paired samples t-tests, showed no statistically significant differences between the right and left sides for any of the eight parameters within the male group or within the female group (p>0.05 for all comparisons). The results of the two-way ANOVA, presented in Table 2, confirmed a statistically significant main effect of sex for every parameter (p<0.001). In contrast, there were no significant main effect of side and no significant interaction effect between sex and side for any measurement.

**Table 2:** Results of the two-way ANOVA analyzing the effects of sex and side on anterior border measurements Two-way ANOVA was used to test the main effects of sex (male, female) and side (right, left) and their interaction effect on each measurement. A significant p-value (<0.05) indicates a significant effect. ANOVA: analysis of variance; ASIS: anterior superior iliac spine; AIIS: anterior inferior iliac spine; PT: pubic tubercle; SS: symphysial surface; IE: iliopubic eminence

Measurement	Effect of sex		Effect of side		Sex-side interaction	
	F-value	P-value	F-value	P-value	F-value	P-value
ASIS-SS	98.45	<0.001	1.892	0.173	0.112	0.739
ASIS-PT	145.62	<0.001	2.456	0.121	0.045	0.832
AIIS-SS	87.33	<0.001	1.234	0.270	0.201	0.655
AIIS-PT	212.89	<0.001	1.567	0.214	0.033	0.856
AIIS-IE	74.11	<0.001	0.789	0.377	0.098	0.755
IE-PT	11.56	0.001	0.445	0.506	1.123	0.292
Arch ASIS-SS	132.67	<0.001	0.892	0.348	1.456	0.231
Arch AIIS-IE	92.34	<0.001	0.334	0.565	0.056	0.814

## Discussion

The findings of this study provide a comprehensive quantitative analysis of the anterior border of the hip bone, confirming robust sexual dimorphism and establishing a notable lack of significant lateral asymmetry in this anatomical region. The finding that all eight measured parameters were significantly larger in males is consistent with the well-documented pattern of general skeletal robusticity in males and aligns with previous morphometric studies focused on the pelvis [7,10]. The results strongly support the work of Sachdeva et al. [7] and Gómez Pellico and Fernández Camacho [6], who identified the anterior border as a key area for sexual discrimination. The high level of significance across all metrics underscores the structural distinctness of this region between the sexes.

The most significant contribution of this study is the detailed investigation into lateral dimorphism. The absence of statistically significant differences between the right and left sides for any parameter, in both males and females, indicates a remarkable degree of bilateral symmetry in the anterior hip border. This finding is crucial as it suggests that the developmental and biomechanical forces shaping this part of the pelvis are highly consistent across both sides of the body. While some studies have reported asymmetry in other pelvic dimensions, often linked to functional dominance [11], our data indicate that the linear distances between the major anterior landmarks are conserved. The two-way ANOVA results further solidify this conclusion, demonstrating that sex is the primary source of morphometric variation, with no significant influence from side or an interaction between sex and side.

The clinical implications of these findings are substantial. The consistent symmetry observed means that in surgical planning, such as for total hip arthroplasty or pelvic fracture repair, the contralateral, unaffected side can reliably serve as a template for reconstructing the injured side [12]. This is particularly valuable for restoring biomechanical parameters like leg length and offset. Furthermore, for forensic anthropologists, the lack of significant asymmetry reinforces the reliability of these measurements; a measurement taken from one side can be confidently compared to population standards without needing a side-specific reference [13]. The provision of detailed mean values and standard deviations for this population offers a valuable baseline for designing prosthetic implants, planning surgical procedures, and interpreting radiographic images in a clinical setting [14].

Limitations

This study has certain limitations. The sample was derived from a specific North Indian population, which may limit the direct generalization of the absolute metric values to other ethnic groups, although the pattern of symmetry is likely a universal anatomical feature. The study was conducted on dry bones; thus, the findings reflect osteological dimensions without the influence of soft tissues, cartilage, or dynamic functional states. Furthermore, the analysis was restricted to linear and arch distances; a geometric morphometric analysis could potentially reveal more subtle shape asymmetries not captured by traditional caliper measurements.

## Conclusions

This morphometric analysis conclusively demonstrates that the anterior border of the human hip bone is a region of significant sexual dimorphism but minimal lateral dimorphism. The consistent bilateral symmetry observed across all parameters enhances the reliability of this anatomical complex for clinical and forensic applications. The data provide a robust reference for the normal anatomical variation of the anterior hip border in the North Indian population, which will aid surgeons in reconstructive procedures and anthropologists in the accurate identification of skeletal remains. Future research should aim to correlate these osteometric data with medical imaging and explore shape-based variations using advanced morphometric techniques.
